# Effects of Gilbert syndrome on cardiovascular disease risk reduction: a systematic review

**DOI:** 10.1186/s43044-026-00738-3

**Published:** 2026-04-20

**Authors:** Nicola Churchill, Luke Barry, Roch Nianogo

**Affiliations:** 1 Department of Epidemiology, University of California, Los Angeles, USA; 2 California Center for Population Research at UCLA, Los Angeles, USA

**Keywords:** Gilbert disease, Public health, Cardiovascular disease, Bilirubin, Humans

## Abstract

**Background:**

Gilbert syndrome is a human genetic disorder which affects bilirubin metabolism in the liver and results in unconjugated hyperbilirubinemia. While considered a benign condition with only occasional jaundice and possible alterations of metabolism of certain drugs, Gilbert syndrome can reduce cardiovascular disease (CVD) risk. The objective of this review was to summarize the evidence on the potential effect of Gilbert Syndrome on reducing CVD risk via lowering cholesterol and/or lipid levels in the body and is associated with other protective mechanisms which are related to higher blood levels of unconjugated bilirubin.

**Methods:**

A systematic review of articles returned from the online database PubMed was conducted using search terms: “Gilbert syndrome”, “reduced cardiovascular disease risk”, “cardiovascular disease”, “cholesterol”, and “lipid level”.

**Results:**

After filtering using the exclusion criteria and removing duplicates, eight articles were identified for the review. This review found that CVD risk was lower for people with Gilbert syndrome when compared to unaffected individuals. The reduced CVD risk was theorized to be due to elevated unconjugated bilirubin levels which lead to reduced concentrations of lipids, reduced inflammation biomarkers, decreased ABCA1 protein, increased serum antioxidant capacity expression, decreased BMI, and lower triglyceride levels.

**Conclusion:**

Individuals with Gilbert syndrome have a reduced CVD risk. Given that Gilbert syndrome reduces CVD risk in individuals and that Gilbert syndrome results in elevated serum levels of unconjugated bilirubin; elevation of unconjugated bilirubin could serve as a biomarker to monitor CVD risk reduction in the general population.

## Introduction

Bilirubin is a normal byproduct of hemolyzed red blood cells and is usually found in the liver. Healthcare workers often test bilirubin levels to assess liver function, red blood cell dysfunction, and gall bladder/bile duct blockage [1]. There are two forms of bilirubin: conjugated and unconjugated. Conjugated bilirubin is usually catabolized in the liver by enzymes, removed from the bloodstream, and excreted in stool after passing into the intestines with bile [2]; showing no impact on cardiovascular diseases (CVD). The process of conjugation renders bilirubin water soluble and increases the size of the molecule, thereby preventing bilirubin from passively being reabsorbed by the intestinal mucosa. Unconjugated bilirubin (UCB) is a compact molecule because of hydrogen bonding and is essentially insoluble in aqueous solutions at neutral pH [3]. Bilirubin is a signaling molecule which, among its activities, protects against reactive oxygen species and acts as a selective peroxisome proliferator-activated receptor α modulator. This allows mice treated with bilirubin to have decreased body weight, body fat, and increased lean body mass [4].

Gilbert syndrome (GS) is a genetic disorder of bilirubin metabolism in the liver resulting in unconjugated hyperbilirubinemia. It does not typically require treatment, has a prevalence of 4–16%, is more commonly found in males, and can include the signs jaundice and reduced capacity to metabolize certain drugs [5, 6]. An important protein in unconjugated stage to a conjugated, water-soluble stage is Uridine diphosphoglucuronate-glucuronosyltransferase 1A1 (UG1A1). When there is a mutation in the promoter region of the gene, UG1A1 is changed to UG1A1*28, making the protein work at 30% capacity [converting bilirubin from an^7^]. This mutation is present in GS and leads to elevated serum levels of UCB. GS is not typically diagnosed until puberty because bilirubin production increases at that stage of development, and males whose parents both carry the modified gene that causes the disorder have a high risk of developing GS [6]. People with hyperbilirubinemia can have lower cholesterol and triacylglycerol concentrations [8]. This can have a substantial effect on cardiovascular diseases because of the known association of higher cholesterol leading to a higher risk of cardiovascular diseases [9].

Cardiovascular diseases (CVDs) are the leading cause of death globally and have many well-known risk factors. Some modifiable risk factors include smoking, physical inactivity, poor diet, excess body fat, high blood cholesterol/lipids, high blood pressure, diabetes, metabolic syndrome, kidney disease, and sleep deficiency [10]. Nonmodifiable risk factors include age, sex, race, and family history. Since the high prevalence of CVD is associated with significant morbidity and mortality, it is important to identify mitigating factors that could reduce CVD risk.

Since GS reduces cholesterol as well as oxidative stress [11, 12], both risk factors for CVD [10], UCB may represent a biomarker for reduced CVD risk. This paper reviews the effect of GS in reducing cardiovascular disease risk.

## Methods

### Search strategy and information source

PubMed database was used for this review. The following search terms were queried in April 2023: “gilbert syndrome”, “cardiovascular disease”, “cholesterol”, and “lipid level”. Individual studies were selected by examining the article’s title, abstract, and full text. When screening full texts, the descriptive statistics of the population and significant findings, methods for study design, population details, statistical methods, and discussion/conclusions for the main findings were gathered and included as elements within Tables 1 and 2.

**Table 1:** Descriptive characteristics of the identified Studies (*N* = 8) of Gilbert Syndrome and Cardiovascular Disease Risk Reduction (Pubmed)

First author and year	Country	Study Design	Population	Exposure	Outcomes	Covariates	CVD risk reduction definition	Gilbert Syndrome definition
Boon (2012) [humans] [11]	Australia	Cohort study of individuals recruited from the general population via internet forums, email adverts and posters.	87 individuals were recruited who were: aged 16–63	Gilberts Syndrome	Thiol and protein oxidation biomarkers and lipid profile	Individuals with GS were matched by age, gender, and BMI with healthy controls.	NA	Previous diagnosis from a medical practitioner, and/or a circulating UCB > 17.1 µmol/L (> 1 mg/dl) and normal serum liver enzyme activities
			Without bacterial or viral infection, antioxidant supplementation, prescribed medication (excl. contraceptive pill)					
			Excessive alcohol consumption (> 8 standard drinks per week)			In regression analysis, LDL, glucose, uric acid and reduced thiols were included to examine model fit but effects were not reported.		
			Elevated glucose or serum liver enzyme activities, lipid metabolism disorder					
			A family history of CVD/hypertension, diabetes, or hematologic or liver disease					
			Did not smoke					
Bulmer (2008) [8]	Australia	Cohort study of individuals recruited from the general population using GS internet forums and public notice forums	A final sample of 21 individuals were recruited from the general population	Gilberts Syndrome	Antioxidant status, oxidative stress and resistance to serum oxidation	Individuals with GS were matched by age, weight and height to healthy controls	NA	General practitioner diagnosis and a circulating UCB concentration of > 17.1 µmol/L (1 mg/dL).
			Without antioxidant supplementation, prescribed medication (excl. contraceptive pill)					
			A family history of CVD, liver disease, renal disease, suffered from chronic illness					
			Excessive alcohol consumption					
			On-smokers					
Kundur (2017) [12]	Australia	Cohort study of volunteers recruited from the general population	A final sample of 28 individuals were recruited who: were non-smokers	Gilberts Syndrome	Platelet activation, aggregation, hemostatic function, lipid status and inflammation	Individuals with GS were matched by age, gender and BMI to controls.	intravascular injury that consequently contributes to thrombus formation	General practitioner diagnosis and/or a circulating UCB concentration of > 17.1 µM
			Had no history of hepatitis, liver diseases, or recent acute respiratory inflammation/bacterial infection			AA-induced platelet aggregation was adjusted for HDL-c		
			No medication or antioxidant consumption 2 weeks prior to participation.			Collagen-induced platelet aggregation was adjusted for HCT (hematocrit), MCV [fL], and BMI [kg/m2]		
			14 individuals with GS were age, gender and BMI matched with 14 control subjects (8 male and 6 female).			ADP [µM] was adjusted for MCV		
			Initial recruitment sample not reported.			P-selectin expression was adjusted for MCH [pg].		
Ocadlik (2011) [19]	Slovakia	Cohort of proband volunteers	100 individuals	Gilberts Syndrome	Atherogenic plasma lipoproteins	Higher triglycerides	NA	• Mutation of the promoter gene for bilirubin-UDP-glucuronosyltransferase (UGT1A1). These individuals had significantly higher total (25.9 vs. 9.7) and unconjugated (16.2 vs. 4.9) billirubin compared to controls.
			Without thyroid disease, liver disease, arterial hypertension, ischaemic heart disease, diabetes mellitus, alcohol and other drug abuse, kidney disease, or malapsorption syndrome			VLDL cholesterol		
						IDL cholesterol		
			40 of these individuals were classified as having GS (see definition).			Small dense LDL levels		
Tapan (2011) [15]	Turkey	Cohort study of patients recruited from the outpatient clinic of the Dept. of Internal Medicine, Gulhane, compared with healthy volunteers.	42 male patients with GS and 50 age, sex and BMI matched healthy volunteers	Gilberts Syndrome	sd-LDL-c (mmol/L), ox-LDL (ng/ml), hs-CRP (mg/L)	GS participants were matched according to age, sex and BMI	NA	Unconjugated hyperbilirubinemia [UCB] (> 17.1 µmol/L) measured on at least two occasions
			No evidence of hemolysis					
			Normal liver enzymes					
			No history of liver disease, diabetes mellitus, hemolysis, hemoglobinopathy, positive hepatitis B surface antigen, anti-hepatitis C virus, drug use in the past week					
			Between age 20 and 25					
			Subjects were from the from the outpatient clinic of the Department of Internal Medicine, Gulhane School of Medicine.					
Vitek (2002) [18]	Czech Republic	Cross-sectional and cohort study of 5 separate groups of participants (A, B, C, D, and E) enrolled from medical centres in Prague.	Group A: 50 participants (35 men and 15 women) with GS and aged > = 40 years, 12 patients lost-to-follow over 3 years	Gilberts Syndrome	IHD, as well as antioxidant status and lipid profile	Probability of developing IHD for groups A and E were estimated in separate models, each adjusting for: Age	Prevalence and incidence of IHD	Diagnosed chronic unconjugated hyperbilirubinemia in the absence of any hemolytic disease and/or any other hepatic function alteration.
			Group B: 38 participants (33 men and 5 women), aged 40–60 years with Ischaemic Heart Disease (IHD)			Sex		
			Group C: 38 healthy participants (14 men and 24 women) aged > 40 years.			Systolic blood pressure	Group A: Symptomatic IHD and ECG	
			Group D: 2296 participants, with similar age and sex characteristics as Group A			Total cholesterol	Group B, C: Confirmed myocardial infarction, or a severe unstable angina pectoris requiring intravenous nitrate therapy	
			Group E: 316 healthy participants			HDL cholesterol		
						Smoking		
						Diabetes mellitus		
						ECG signs for left ventrocular hypertrophy	Group E: Not specified.	
Wallner (2013) [humans] [16]	Austria	Cohort study of individuals recruited from the general Austrian population	A final sample of 118 individuals were recruited from the general Austrian population	Gilberts Syndrome	Lipid profile and adiposity		NA	Fasting serum UCB concentration of ≥ 17.1 µmol/L.
			Non-smokers			Individuals with GS were matched by age, and gender with healthy controls		
			Aged 20–80 years			Stratification according to age (< 30 vs. >=30)		
			Without liver, heart or kidney disease, haemolysis, diabetes, cholelithiasis, organ transplants, history of CVD, cancer, high alcohol consumption, excessive physical activity, vitamin supplementation, and prescribed medication (excl. contraceptive pill)					
			9 subjects, identified as GS					
Wang (2017) [humans] [17]	Austria	Cohort study of blood samples from recruited patients	60 patients with Gilberts syndrome	Gilberts Syndrome	Cholesterol efflux mediated by plasma	These variables were included to improve the model but effects were not reported: HDL cholesterol	NA	Fasting serum UCB concentration of ≥ 17.1 µmol/L.
			60 age- and sex- matched healthy control subjects.			BMI		
						Age		
						Sex		

*Acronym: GS = Gilbert syndrome, CVD = cardiovascular disease, BMI = body mass index, LDL = low-density lipoprotein, UCB = unconjugated bilirubin, AA = arachidonic acid-induced platelet aggregation, HDL-c = high-density lipoprotein cholesterol, HCT = hematocrit, MCV = mean cell volume, ADP = adenosine diphosphate, MCH = mean cell hemoglobin, VLDL = very low density lipid, IDL = intermediate-density lipoprotein, sd-LDL-c = small dense LDL cholesterol, ox-LDL = oxidized LDL, hs-CRP = high sensitivity C-reactive protein, IDH = ischemic heart disease, ECG = electrocardiogram, ANOVA = one-way analysis of variance, HDL = high-density lipoprotein, TAG = triacylglycerols, AA = arachidonic acid

**Table 2:** Statistical characteristics of the identified Studies (*N* = 8) of Gilbert Syndrome and Cardiovascular Disease Risk Reduction (Pubmed)

First author and year	Statistical methods	Results	Bias score	Conclusion
Boon (2012) [humans] [11]	Two-tailed unpaired students t-test, Mann-Whitney rank sum test, Pearson correlation, forward step-wise regression, Alpha = 0.05.	Forward stepwise regression analysis revealed that bilirubin was associated with increased reduced glutathione: oxidized glutathione (GSH: GSSG) ratio and reduced thiol concentrations, which, in addition to reduced circulating LDL, probably decreased oxLDL concentrations within the cohort.	7	Elevated circulating bilirubin is associated with improved circulating thiol and lower cholesterol which may explain part of the relationship between GS and atherosclerosis.
Bulmer (2008) [8]	Unpaired t-tests and Rank Sum tests, Pearson correlation, Alpha = 0.05	The lag phase of serum oxidation was significantly longer in the GS group (GS: 121.4 ± 10.5; control: 106.8 ± 14.6 min; *P* = 0.020) and was positively correlated with the bilirubin concentration (*r* = 0.451, *P* = 0.040). A trend toward elevated HDL: LDL ratio was observed in GS	6	Resistance to serum oxidation and increased circulating antioxidant status is related to elevated UCB among individuals with GS and may help offer protection against CVD
Kundur (2017) [12]	Power calculations, Unpaired t-tests, Correlation, Forward step-wise regression, Alpha = 0.05.	Individuals with GS had elevated red blood cell (RBC) mean cell volume (MCV), mean cell hemoglobin (MCH), and mean corpuscular hemoglobin concentration (MCHC) in comparison with control individuals. Bilirubin, by itself, was able to explain the variations in AA (37%), collagen (44%), and ADP (15%)-induced platelet aggregation	7	In addition to the observed association of GS with lipid and inflammation biomarkers, elevated bilirubin among GS subjects may also reduce CVD risk via reduced thrombosis from inhibition of platelet aggregation and granule release.
Ocadlik (2011) [19]	Pearson and spearman correlation, student’s t-test for unpaired observations. Alpha = 0.05.	In the exposed group, we found significantly negative correlation between serum unconjugated bilirubin levels and LDL 3–7 (seven subfractions of LDL), as well as between bilirubin and triglycerides. Serum bilirubin concentration and LDL 1–2 (two subfractions of LDL) concentration correlated significantly positively. In the unexposed group, there was significant negative correlation between serum bilirubin levels and LDL 3–7, too.	4	GS is associated with a low occurrence of strongly atherogenic small dense LDL which may have a protective effect against atherosclerosis
Tapan (2011) [15]	Kolmogorov-Smirnov tests, Levene’s tests, t-test and Mann-Whitney U-tests, Spearmans correlation, Multivariate linear regression, Alpha = 0.05.	UB were negatively correlated with small dense low density lipoprotein cholesterol (sd-LDL- C), oxidized LDL (ox-LDL) and high sensitivity C-reactive protein (hs-CRP), and sd-LDL-C was positively correlated with ox-LDL	4	Circulating levels of sd-LDL-c, ox-LDL, and hs-CRP are lower among individuals with GS compared to healthy controls, suggesting a potential pathway by which bilirubin may prevent atherosclerosis.
Vitek (2002) [18]	Kolmogorv-Smirnov test, 1-way ANOVA, Scheffe method, Fisher linear discriminant analysis, Chi-squared tests, Regression analysis, Poisson probability distribution, Alpha = 0.05	Linear discriminant analysis used as a technique to assess the preventive role of UCB and HDL levels on the development of IHD revealed a positive correlation between elevated bilirubin levels and a lower incidence of IHD	4	Lower prevalence of IHD was found between GS patients and the general population. Chronic hyperbilirubinemia prevented the development of IHD by increasing the serum antioxidant capacity.
Wallner (2013) [humans] [16]	Kolmogorov-Smirnov tests, Students t-test, Mann-Whitney U-tests, ANOVA, Kruskal-Wallis H test, Student-Newman-Heuls post-hoc tests, Pearsons and Spearmans correlation, forward step-wise regression, Alpha = 0.05.	When analyzing the entire cohort significant negative correlations between UCB and BMI (*r* = − 0.211, *P* < 0.05), total cholesterol (*r* = − 0.245, *P* < 0.01), LDL-C (*r* = − 0.243, *P* < 0.01), TAG (*r* = − 0.248, *P* < 0.01), LDL-1 (*r* = − 0.247, *P* < 0.05), LDL-2 (*r* = − 0.272, *P* < 0.05) and the low-atherogenic subfractions (*r* = − 0.276, *P* < 0.05) were found. Forward stepwise regression analysis revealed that bilirubin explained 21% of the variance in LDL-C, 15% of total cholesterol, 4.5% of LDL-1 and 4% of LDL-C in GS subjects	7	GS is associated with reduced concentrations of lipid and inflammation biomarkers and a trend towards lower adiposity (but not significant). Clearer differences were generally observed between older subjects with and without GS, and between younger and older controls suggesting GS may delay age-related changes in these outcomes and potentially CVD.
Wang (2017) [humans] [17]	2-tailed unpaired Student t-test, paired t test, 1‐way ANOVA, Bonferroni post hoc test, forward step-wise regression, Alpha = 0.05.	Hyperbilirubinemic plasma from patients with Gilbert syndrome and Gunn rats induced significantly reduced cholesterol efflux compared with normobilirubinemic plasma. Unconjugated bilirubin (3–17.1 µmol/L) exogenously added to plasma- or apolipoprotein A1–supplemented media also decreased macrophage cholesterol efflux in a concentration‐ and time‐dependent manner	5	Cholesterol efflux mediated by plasma is significantly lower among humans with GS, suggesting a role in CVD outcomes via metabolism.
All reported effects were found to be statistically significant at the reported threshold (alpha) for each study				

*Acronym: GS = Gilbert syndrome, CVD = cardiovascular disease, BMI = body mass index, LDL = low-density lipoprotein, UCB = unconjugated bilirubin, AA = arachidonic acid-induced platelet aggregation, HDL-c = high-density lipoprotein cholesterol, HCT = hematocrit, MCV = mean cell volume, ADP = adenosine diphosphate, MCH = mean cell hemoglobin, VLDL = very low density lipid, IDL = intermediate-density lipoprotein, sd-LDL-c = small dense LDL cholesterol, ox-LDL = oxidized LDL, hs-CRP = high sensitivity C-reactive protein, IDH = ischemic heart disease, ECG = electrocardiogram, ANOVA = one-way analysis of variance, HDL = high-density lipoprotein, TAG = triacylglycerols, AA = arachidonic acid

## Eligibility criteria

Studies were excluded if they were systematic reviews or review papers. Commentary papers on previous studies were also excluded. Studies that did not involve the exposure of interest (GS) or the outcome of interest (reduced CVD risk or risk factors) were deemed ineligible. Drug trials were also excluded.

Studies that had a primary focus of GS as the exposure and CVD as the outcome were included. All studies included focused on GS as the exposure, and this was usually confirmed with a previous diagnosis or UCB concentration of > 17.1 micro-mol/L. Studies that looked at humans with GS were included.

## Data extraction and study quality assessment

Full-text review was carried out by two reviewers in duplicate (NC and LB). Any disagreements would be resolved by a third reviewer (RN). Valid findings and characteristics of each paper were gathered into summary tables (see Tables 1 and 2). These variables included first author (last name followed by year of publication), country, study design, population characteristics for people included in the study, exposure, outcome (and mechanism, if given), covariates included in regression models, CVD reduction definition (if available), GS definition, statistical methods, results, risk of bias, and conclusions.

Bias of studies was assessed by one reviewer (NC) using the following criteria: appropriateness of study design and sample size for addressing the research objectives, generalizability, participant or condition selection methods, response and attrition rate, measurement of study variables, control of confounding, appropriateness of statistical analyses, quality of reporting, quality of intervention/condition, and authors’ conflict of interest [13]. These criteria were used to create a zero to ten scale where zero was the most bias and ten was the least. Each criterion was scored zero or one and added to create the total bias score. Appropriateness of study design and sample size were marked as adequate if there were 100 or more participants in the study, combining exposed and unexposed individuals. The generalizability was considered if the sample represented their target population. For participant or condition selection methods, the reviewers focused on how the participants were recruited for the study. The response and attrition rates were selected by seeing who participated in the study versus who was recruited. It should be noted that some studies only stated who participated and did not refer to the recruitment technique or how many were recruited; these studies received a 0 in that category if they did not supply any details on the recruitment process. The measurement of study variables was determined by the process of collecting the data, which was mainly blood samples in this case. For confounding control and appropriateness of analyses, reviewers looked at the regression analysis description and results. The quality of intervention focused on how GS was defined. Lastly, the authors’ conflict of interest was inspected by looking at the funding and acknowledgment section of each paper.

## Results

### Study selection

**Fig. 1:**
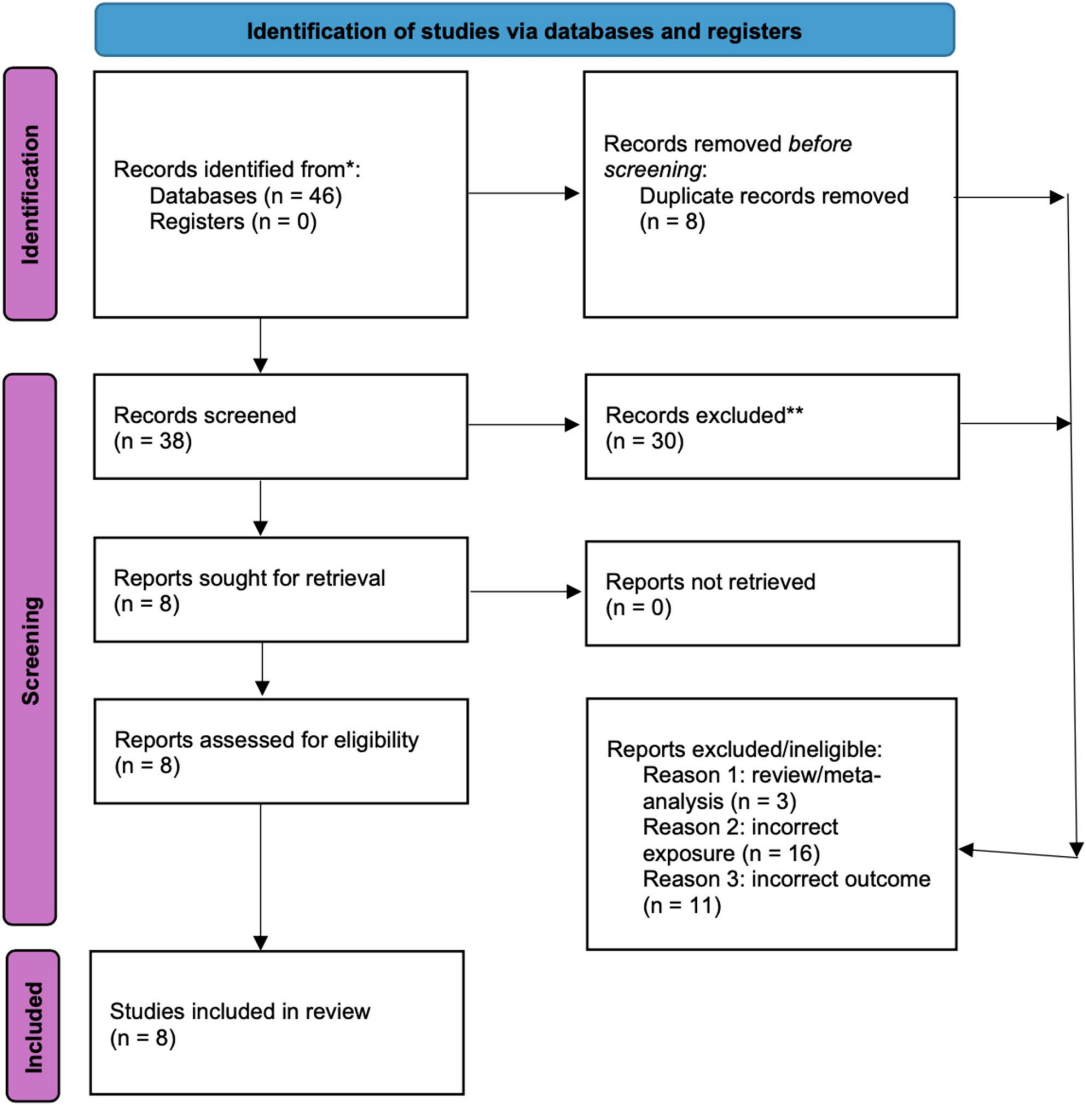
PRISMA 2020 flow diagram for new systematic reviews which included searches of PubMed

Using the search terms discussed above, we organized the study selection using the 2020 PRISMA diagram format (Fig. 1**)**. There were 46 research articles collected, of which eight were duplicates. After removing duplicates, 38 articles were screened and 30 were excluded for one of three reasons: paper was a review/meta-analysis (*n* = 3), incorrect exposure, (*n* = 16), or incorrect outcome (*n* = 11). Incorrect exposure or outcome were defined as any exposure or outcome other than GS or CVD/a specific type of CVD, respectively; leaving eight studies eligible and included in this review.

### Study year, country, and study design

Eligible papers were published 2002–2017 from Australia, Slovakia, Turkey, Czech Republic, and Austria. All studies took place in Europe, likely because GS is more common in Europe [14]. Most studies were matched cohort studies (*n* = 7) where people were grouped into those with GS and those without. Matches were usually by age, sex, and BMI but one study matched on age, sex, height, and weight and two matched on just age and sex [8, 11, 12, 15, 16].

## Study population, exposure, outcome, and covariates

Most papers included subjects who were nonsmokers, did not excessively drink, did not take antioxidant supplementation, and did not take prescribed medication. Other studies avoided people with family history of CVDs or other diseases such as diabetes, liver disease, hypertension, and kidney disease. Selected subjects for both exposed and unexposed groups were generally healthy. Outcomes included thiol oxidation; markers of antioxidant and oxidative stress; susceptibility of serum to oxidation; thrombotic risk factors including platelet activity, hemostatic function, and inflammation; occurrence of atherogenic plasma lipoproteins; sd-LDL-C, ox-LDL and high sensitivity C-reactive protein (hs-CRP) levels; ischemic heart disease; well-accepted biomarkers of lipid metabolism and adiposity and also novel lipid-associated CVD risk markers; and macrophage cholesterol efflux. Common covariates included in regression models of the eligible papers include age, Body Mass Index (BMI), glucose, total cholesterol, triglycerides, high density lipid (HDL), low density lipid (LDL), UCB, alanine aminotransferase, aspartate aminotransferase, and gamma-glutamyltransferase (see Table 1). However, many studies used adjustment to examine whether UCB provided additional explanatory power beyond selected covariates in explaining differences in CVD outcomes or risk factors, without reporting effect sizes (e.g. examining the R-squared only). Thus, the effect sizes reported for these studies are the crude/unadjusted estimates [11, 12, 16, 17].

## CVD definition

Each included study stated that their purpose was to examine the relationship between GS and CVD. A definition of CVD risk reduction was often omitted in these papers, except for two studies. It was defined as intravascular injury, consequently contributing to thrombus formation in the study done by Kundur [12]. Platelet aggregation or the diagnosis of ischemic heart disease (IHD) based on past medical history of symptomatic IHD, (occurrence of stenocardia, positive effect of nitrate therapy) and ECG criteria (specific changes of ST segment, presence of Q wave, incidental left bundle branch block) was used to define CVD in the study conducted by Vitek [18].

### Gilbert syndrome definition

GS was usually defined as possessing a previous diagnosis from a medical practitioner and/or having a circulating UCB concentration ≥ 17.1 µM (≥ 1 mg/dl) [11, 12, 15–17]. One definition included having normal serum liver enzyme activity in addition to the two aforementioned more common criteria [11]. One paper defined GS based on molecular-genetic testing for the presence of mutation in the promoter gene for bilirubin-UDP-glucuronosyltransferase (UGT1A1) [19]. Another defined GS as chronic unconjugated hyperbilirubinemia in the absence of any hemolytic disease and/or any other hepatic function alteration [18].

### Statistical methods

A variety of statistical methods were used in eligible studies. The most common techniques used were unpaired t tests, forward stepwise regression, Pearson and Spearman correlation, Kolmogorov Smirnov test, and ANOVA. Other analyses used were Levene’s test, Bonferroni post hoc test, Kruskal–Wallis H test, Student–Newman–Keuls post-hoc tests, Mann–Whitney U test, and Scheffe method of multiple comparisons. All studies used descriptive statistics (see Table 2).

### Study results

The valid results of the eight studies included were distinctive to each study. All reported effects were found to be statistically significant at the reported threshold (alpha = 0.05). The main results were that UCB was associated with lower GSH: GSSG ratio and reduced thiol concentrations which probably decreased oxidized low-density lipoprotein (LDL) concentrations [11]; serum oxidation in the GS group was positively correlated with the bilirubin concentration (correlation coefficient [r] = 0.451, p-value [p] = 0.04) and a trend toward elevated high-density lipoprotein: low-density lipoprotein (HDL: LDL) ratio was observed [8, 12]; bilirubin, by itself, was able to explain the variations in arachidonic acid (AA) (37%), collagen (44%), and adenosine diphosphate (ADP) (15%)-induced platelet aggregation; in the group with GS, a significantly negative correlation was found between serum UCB levels and LDL 3–7 (seven subfractions of LDL), as well as between bilirubin and triglycerides and serum bilirubin concentration and LDL 1–2 (two subfractions of LDL) concentration correlated significantly positively [19]; UCB was negatively correlated with small dense LDL cholesterol (sd-LDL-c), oxidized LDL (ox-LDL) and high sensitivity C-reactive protein (hs-CRP), and sd-LDL-c was positively correlated with ox-LDL [15]; linear discriminant analysis used as a technique to assess the preventive role of UCB and HDL levels on the development of IHD revealed a positive correlation between elevated bilirubin levels and a lower incidence of IHD [18]; when analyzing the entire sample significant negative correlations between UCB and body mass index (BMI) (*r* = − 0.211, *p* < 0.05), total cholesterol (*r* = − 0.245, *p* < 0.01), LDL-c (*r* = − 0.243, *p* < 0.01), triacylglycerols (TAG) (*r* = − 0.248, *p* < 0.01), LDL-1 (*r* = − 0.247, *p* < 0.05), LDL-2 (*r* = − 0.272, *p* < 0.05) and the low-atherogenic subfractions (*r* = − 0.276, *p* < 0.05) were found [16]; and hyperbilirubinemic plasma from patients with GS induced significantly reduced macrophage cholesterol efflux compared with normobilirubinemic plasma and UCB (3–17.1 µmol/L) exogenously added to plasma- or apolipoprotein A1–supplemented media also decreased macrophage cholesterol efflux [17] (Table 2).

### Bias scores

There were four studies that achieved bias scores that were greater than five, which is considered minimum bias. The other four studies had scores of five or less, resulting in a substantial bias score. The less biased papers showed generalizability, appropriate participant selection, well-measured variables, appropriate statistical analysis, quality of intervention, and no conflicts of interest. Four papers lacked strength in the following areas: generalizability, participant selection, attrition rate, control of confounding, and quality of reporting. The weaker papers tended to have less description in their methods and results.

### Study interpretations

The eight papers included had similar conclusions despite their differing mechanisms of determining CVD risk reduction. The study by Boon, et al. (2012), concluded that elevated circulating bilirubin was associated with improved circulating thiol status and hypocholesterolemia, which could protect from atherosclerosis in patients with GS [11]. Bulmer, et al. (2008), concluded individuals with GS have an increased circulating antioxidant status and improved resistance to serum oxidation, which may partially explain their reduced prevalence of CVD [8]. A study done by Kundur, et al. (2017), stated elevated levels of circulating bilirubin may be associated with reduced risk of thrombosis by inhibition of platelet aggregation and granule release in GS patients [20]. Ocadlik, et al. (2011), found that patients with GS had significantly lower serum levels of small dense LDL, very low-density lipoprotein (VLDL) and triglycerides. The same authors also found hyperbilirubinemia in persons with GS has an anti-atherogenic effect and is enhanced by a presence of non-atherogenic lipoproteins [19]. Tapan, et al. (2011), found small dense LDL cholesterol (sd-LDL-C), a well-known mediator of initial stages of atherosclerosis, was lower in subjects with GS when compared to healthy controls and that oxidized LDL (ox-LDL) and high sensitivity C-reactive protein (hs-CRP) levels were also decreased in those patients with GS [15]. In addition, there were significant negative correlations of UCB with sd-LDL-C and ox-LDL. Vitek, et al. (2002), stated chronic hyperbilirubinemia prevented the development of ischemic heart disease by increasing the serum antioxidant capacity [18]. The study conducted by Wallner, et al. (2013), stated benign, elevated circulating bilirubin is associated with reduced concentrations of lipid and inflammation biomarkers and a trend toward decreased BMI [16]. This study also communicated the unique finding that older subjects are likely to benefit more from a mild congenital hyperbilirubinemia. Lastly, Wang, et al. (2017), most recently demonstrated a direct effect of UCB on reduced macrophage cholesterol efflux and an association with decreased ABCA1 protein expression [17].

## Discussion

We reviewed studies investigating the effect of GS on CVD to extend the literature related to the last review paper on this subject published in March 2015 [20]. From 38 papers initially identified, eight studies were included for full review after screening and applying the inclusion criteria. Our review suggests that GS has a protective effect against cardiovascular diseases due to its resultant higher levels of UCB. This aligns with the conclusions from all eight studies included in this review and the previously conducted systematic reviews.

The general theme of the selected studies is people with GS have different biologic risk factors that protect them from CVD through a variety of mechanisms. The definition of GS in all studies was generally uniform throughout different studies which made comparisons between exposed groups in different studies possible. The generality with which CVD was defined made conclusions and comparisons between studies difficult. The two studies that did define CVD explicitly made their conclusions and goals of the study more interpretable [12, 18]. However, the utilized study design had a major impact on bias and quality of studies. The papers with the lowest amount of bias concluded that GS was associated with (i) an increased reduced GSH: GSSG ratio and (ii) a reduced thiol concentration [11]. In addition, individuals with GS had (i) elevated red blood cell (RBC), (ii) mean cell volume (MCV), (iii) mean cell hemoglobin (MCH), and (iv) mean corpuscular hemoglobin concentration (MCHC) in comparison with control individuals [11, 12]. In other words, people with GS had more protective biologic factors against CVD than people without the disease.

GS patients have a reduced CVD risk likely due to: (i) the effect of UCB in decreasing biomarkers of CVD such as lipid levels, and (ii) the increasing protective factors against CVD such as serum antioxidant capacity expression. The theorized mechanisms for reducing CVD risk include several known factors associated with reduced CVD, though the certainty that GS reduces CVD risk through these factors has not been proven at the time of this review. Given that GS reduces CVD risk in individuals and that GS results in elevated serum levels of UCB; elevated UCB at a certain defined threshold may serve as a biomarker for reduced CVD risk in the general population. There are several benefits to considering UCB as a biomarker. High UCB levels inversely correlate with CVD risk. Tests for bilirubin, both conjugated and unconjugated, are routinely performed on patients as part of a normal screening panel of serum tests and the test for bilirubin is inexpensive and has remained unchanged for several decades. These latter two factors would allow for retrospective review of electronic health databases to examine various threshold levels of UCB in patients and finding which of those patients go on to develop CVD or are protected from development of CVD compared to the lower level UCB patients. Electronic heath records studies usually involve large sample sizes, helping to avoid bias due to small numbers; a common bias noted amongst studies in this review. In this way, a CVD risk stratification of UCB levels could be elucidated, a well-defined threshold of what level of UCB can result in reduced CVD risk could be achieved, and UCB could be used as a biomarker of reduced CVD risk.

Additionally, UCB’s beneficial physiologic effects could present a model for pharmacological management of biomarkers for CVD risk reduction. The pharmacologic exploration of bilirubin is currently under research and is being tested in mice models. When mice were injected with bilirubin, they exhibited a fourfold increase in total plasma bilirubin, and this resulted in reduced plasma glucose, total cholesterol, and LDL cholesterol levels. This new research has also found that lesions that were present throughout the aorta were resolved in mice that had been treated with bilirubin and the mice also had decreased size and lipid load of the aortic sinus plaque and aortic sinus lesion area [17]. With many positive results already shown in mice, the next step is developing pharmacological treatments which might increase endogenous UCB or create medications which can be similar enough to the UCB molecule and be active in reducing the aforementioned biomarkers. A recent Australian study looked at the effect of silymarin on bilirubin levels, lipid status, systemic inflammation, and antioxidant status. Their results showed no impact on bilirubin status, lipid status, systemic inflammation, or antioxidant status; providing a caution against the use of silymarin to cause hyperbilirubinemia and its associated beneficial effects [21]. While therapies that may increase UCB may be a long way in the future in development, a new class of medications would add to the current armamentarium for the treatment of CVD and defining UCB as a biomarker would streamline research for CVD risk reduction as it affects several known biomarkers and increases protective factors and decreases risk factors for CVD.

Our study is not without limitations. First, the included studies in general had small sample sizes which could reduce power to detect associations. In fact, larger matched cohort studies are difficult and time-consuming to conduct if they are done with adequate number of subjects reinforcing the potential value of retrospective studies using electronic health records. Second, we focused only on human results even where murine results were provided within the same study in order to ensure the highest relevance to human populations. As a result, we ignored some interesting conclusions made about the rat models that could be relevant with regards to genetic results. For example, mice results found a higher UCB was associated with lower mass, cholesterol, HDL and HDL: LDL; UCB explained 45% of the variance in total cholesterol, 42.4% in HDL-c and 28.3% in TAG; and No association between UCB and cholesterol efflux mediated by plasma in a best fit model [11, 16, 17]. In general, there is a significant lack of research in GS and it’s benefits to CVD in humans.

## Conclusion

This review revealed that there is an association between high UCB levels and reduced risk of CVD. Given this finding, unconjugated bilirubin should be considered when planning future treatment for CVD and for biomarker consideration for CVD risk. Treatment through raising one’s unconjugated bilirubin could be a possible new therapy for individuals with a higher risk of CVD. This potential biomarker for protection could better identify high risk individuals and focus resources on the highest risk group. A potential risk for this possible treatment is that there is little known on the side effects of increasing a person’s UCB with the purpose to decrease their CVD risk. There is still more research to be done on GS and CVD, and larger, more generalizable cohort studies can impact the current literature.

## Data Availability

No datasets were generated or analysed during the current study.

## Abbreviations

GS: Gilbert syndrome
CVD: Cardiovascular disease
BMI: Body mass index
LDL: Low-density lipoprotein
UCB: Unconjugated bilirubin
AA: Arachidonic acid-induced platelet aggregation
HDL-c: High-density lipoprotein cholesterol
HCT: Hematocrit
MCV: Mean cell volume
ADP: Adenosine diphosphate
MCH: Mean cell hemoglobin
VLDL: Very low density lipid
IDL: Intermediate-density lipoprotein
sd-LDL-c: Small dense LDL cholesterol
ox-LDL: Oxidized LDL
hs-CRP: High sensitivity C-reactive protein
IDH: Ischemic heart disease
ECG: Electrocardiogram
ANOVA: One-way analysis of variance
HDL: High-density lipoprotein
TAG: Triacylglycerols
AA: Arachidonic acid
